# Impact of the intensive psychological intervention care on post-traumatic stress disorder and negative emotions of teenage female patients seeking an induced abortion

**DOI:** 10.3389/fpsyt.2023.1033320

**Published:** 2023-10-12

**Authors:** Huiling Liu, Fengdi Wu, Guixia Liao, Sizi Mai, Meijin Ouyang

**Affiliations:** ^1^Department of Gynecology, Shunde Hospital, Southern Medical University (The First People’s Hospital of Shunde), Foshan, China; ^2^Nursing Department, Shunde Hospital, Southern Medical University (The First People’s Hospital of Shunde), Foshan, China; ^3^Department of Clinical Psychology, Shunde Hospital, Southern Medical University (The First People’s Hospital of Shunde), Foshan, China

**Keywords:** intensive psychological intervention care, induced abortion, adolescent, PTSD, anxiety, depression

## Abstract

**Aim:**

This study aimed to investigate the effects of intensive psychological intervention care on adverse emotions and post-traumatic stress disorder (PTSD) symptoms in female teenage patients after induced abortion.

**Methods:**

This prospective cohort study included 100 teenage female patients seeking induced abortion who were randomly divided into two groups: the intervention group (*n* = 50) and the control group (*n* = 50). The intervention group received intensive psychological intervention care, while the control group received standard routine nursing. The scores of the PTSD checklist for DSM-5 (PCL-5), self-rating depression (SDS), and self-rating anxiety scale (SAS) were compared between the two groups at 1 month and 3 months after the operation.

**Results:**

The intervention group had lower risk of developing PTSD (24% vs. 44%), depression (10% vs. 32%), and anxiety (0% vs. 12%) symptoms at 1 month after the surgery. However, there were no significant differences observed between the two groups at 3 months after the surgery. Furthermore, the intervention group had significantly lower scores in PCL-5 (27.4 ± 5.4 vs. 31.8 ± 5.7; 20.5 ± 7.1 vs. 25.0 ± 7.5; *p* < 0.05), SDS (31.8 ± 5.4 vs. 37.8 ± 6.6; 26.8 ± 5.0 vs. 31.4 ± 7.2; *p* < 0.05), and SAS (32.7 ± 5.0 vs. 39.8 ± 6.9; 25.0 ± 2.7 vs. 27.5 ± 2.8; p < 0.05) at 1 month and 3 months after induced abortion.

**Conclusion:**

These findings suggest that intensive psychological intervention care can reduce the incidence and severity of depression, anxiety, and PTSD symptoms in teenage patients who undergo induced abortion.

**Clinical trial registration:**

https://www.chictr.org.cn/showproj.html?proj=185200, identifier ChiCTR2300067531.

## Introduction

Globally, the high rates of induced abortion among young women are a significant public health concern (1). In the United States, nearly 1 million women seek induced abortion each year, with 37% of them being under the age of 25 in 2018 (2). Similarly, in China, 47.5% of women seeking induced abortion were under the age of 25 (3, 4). A study conducted in Singapore revealed that 9.1% of women seeking induced abortion were under the age of 20 (5). Compared to women over the age of 20, younger women are more likely to undergo abortion due to limited knowledge about reproduction and contraception, as well as lack of access to post-abortion care services. Induced abortion during adolescence increases the risk of adverse pregnancy outcomes in later pregnancies, such as anemia, stillbirth, premature delivery, and low birth weight infants (6). The experience of induced abortion can have long-term physical and psychosocial impacts on women, particularly for those aged 15–18, who may struggle to fully cope with the clinical and social consequences (7).

Psychological sequelae resulting from induced abortion have long been a topic of concern among researchers (8). Posttraumatic stress disorder (PTSD) or posttraumatic stress symptoms (PTSS) are highly prevalent in the general female population due to genetic susceptibility, personality factors, and ongoing mental stress (9). A cross-sectional study conducted in Sweden revealed that that the lifetime prevalence rate of PTSD in women who underwent induced abortion was 7%, with a point prevalence rate of 4%. Additionally, the prevalence rate of PTSS during the abortion procedure was found to be 23% (10). Another Swedish multi-centre cohort study discovered that the lifetime prevalence of PTSD in women who had undergone induced abortion was 7.2% at baseline, 2.9% at 3 months, and 2.3% at 6 months. It is worth noting that among females aged 15 to 19, 42.1% (24/57) experienced PTSD or PTSS during post-abortion observation, compared to 28.8% (197/684) in women over 20 years of age (11). Furthermore, a large epidemiological study demonstrated that women with PTSD had an increased risk of ectopic pregnancy, spontaneous abortion, premature delivery, and fetal overgrowth (12). Effective management of PTSD can be achieved through psychological and pharmacological interventions (13, 14). To reduce or prevent the occurrence of PTSD, it is important to provide psychological intervention to adolescent patients who have undergone induced abortion.

Multiple studies have shown that anxiety and depression are risk factors for PTSD (15). Additionally, there is evidence suggesting that anxiety and depression often coexist with PTSD in the majority of cases (16). It is clear that depression and anxiety have negative effects on women after abortion, both at an individual and social level. A longitudinal single-arm cohort study found that there is no significant difference in the incidence of depression and PTSD between postpartum and induced abortion patients. At 3 months after delivery/abortion, 6.3 and 11.1% of pregnant women experienced mild and severe depression, respectively (17). In a study comparing the mental health of women who had undergone induced abortion with who had not, it was found that the former group experienced worse outcomes in terms of anxiety, self-esteem, life satisfaction, and emotions (18).

Previous research has primarily concentrated on the occurrence and risk factors of teenage mental health issues following induced abortion, with limited exploration on prevention or treatment. Teenagers who experience post-traumatic stress disorder (PTSD) after induced abortion exhibit distinct pathophysiological characteristics compared to adults, highlighting the need for enhanced psychological interventions.

## Methods

This prospective cohort study enrolled 100 female teenage patients who underwent induced abortion and were admitted to Shunde Hospital of Southern Medical University from June 2021 to September 2021. The patients were randomly divided into two groups: the control group (*n* = 50) and the intervention group (*n* = 50). Ethics approval (20201216) was obtained from the ethics committee of Shunde Hospital, Southern Medical University (The First People’s Hospital of Shunde). Written informed consent was obtained from all patients.

**Figure 1:**
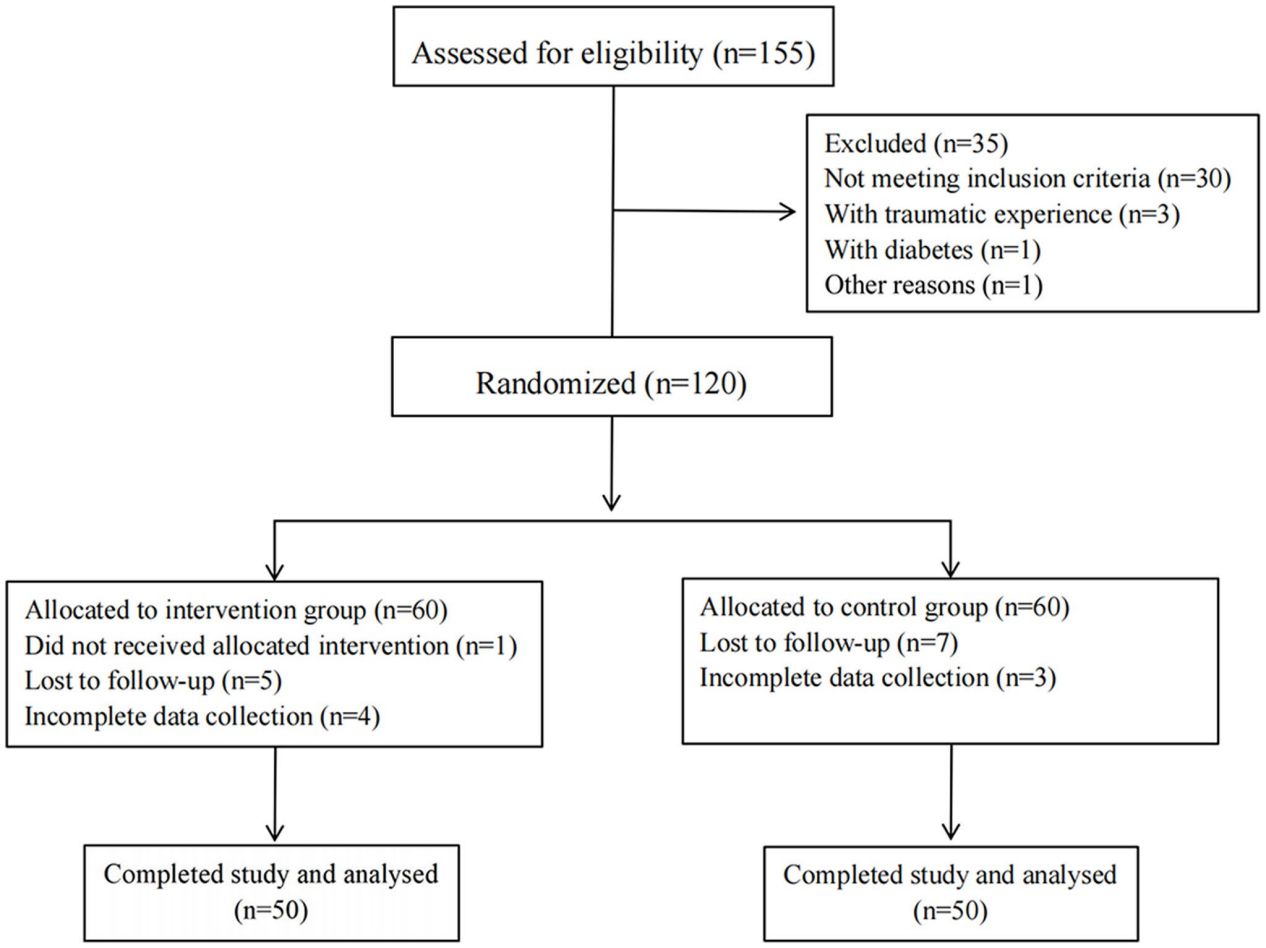
A CONSORT flow diagram outlining the study protocol.

The study consort diagram is shown in Figure 1.

### Inclusion/exclusion criteria

#### Inclusion criteria

The study included pregnant teenagers aged 14–19 years old who came to the hospital seeking abortion and had a gestational age within 12 weeks.

#### Exclusion criteria

(1) Severe underlying diseases such as diabetes, hypertension, and cancers; (2) mental health problems; (3) traumatic experiences in the past year (physical or sexual abuse, natural disasters, accidents, etc.); and (4) inability to complete the follow-up.

### Interventions

The control group patients received routine nursing care, which included medical services for complications of abortion, post-abortion family planning services (PAFPS), counseling services, community services, and comprehensive reproductive health services.

Intervention group patients received intensive psychological intervention care through phone interviews. This included one-on-one stress support treatment, which was conducted four times (once a week, starting from the first week after the abortion). The treatment incorporated various cognitive behavioral therapies such as emotional expression, normalization, psychological education, and cognitive re-evaluation. Each session lasted approximately 30 min and was conducted by the same trained nurse. Throughout the treatment, patients in the intervention group were encouraged to utilize relaxation and recovery techniques, such as relaxation music and meditation exercises.

### Measurement

Psychological conditions were assessed using the PTSD Checklist for DSM-5 (PCL-5), the self-rating anxiety scale (SAS), and the self-rating depression scale (SDS) at 1 month and 3 months after the induced abortion.

For PCL-5, individuals assess the severity of 20 symptoms on a five-point scale (0 = none at all, 4 = very serious), and a reasonable cut score is 33. Symptom severity of PTSD was generally higher with a higher score (19).

The Self-rating Depression Scale (SDS) is composed of 20 items and has a cutoff value of 53 points. If the SDS score is ≤52, there are no signs of depression. A score of 53–62 indicates mild depression, 63–72 indicates moderate depression, and ≥73 indicates severe depression (20).

The SAS score is calculated based on the scores of 20 items, with each item scored between 0 and 4 points. The SAS scores are cut off at 50 points, and a higher SAS score indicating greater risk of anxiety (21, 22).

### Statistical analysis

Based on previous interviews, the estimated PTSD prevalence of teenage patients seeking an induced abortion was 50%. To ensure statistical accuracy, bilateral *α* was set at 0.05 and power was set at 80%. Additionally, a loss of follow-up and rejection rate of 10–20% was taken into consideration. Therefore, a total of 50 participants are required for each group in the trial.

The data analysis and processing were performed using SPSS 21.0 software. For continuous variables that followed a normal distribution, mean and standard deviation (SD) were calculated, and an independent *t*-test was conducted. For categorical variables and continuous variables that did not follow a normal distribution, the Mann–Whitney U test or chi-square test was employed. A value of *p* of 0.05 was considered statistically significant.

## Results

### Demographic information of the participants

Intervention and control groups consisted of individuals aged between 15–20 years old. There were no significant differences in age, gestational weeks, education, and family structure between the two groups, as shown in Table 1.

**Table 1:** Sociodemographic characteristics of 100 teenage female with induced abortion.

	Intervention group (*n* = 50)	Control group (*n* = 50)	*p*-value
Age	18.67 ± 0.85	18.74 ± 1.02	0.727
Gestational weeks	6.74 ± 3.20	6.32 ± 1.63	0.226
Education			0.814
Senior high	18	15	
Junior high	24	26	
Dropout	8	9	
Family structure			0.798
Two biological parents	14	12	
Other two parent	21	20	
Single parent	15	18	
Abortion methods			0.656
Negative pressure suction	35	37	
Forceps curettage	15	13	

### Comparison of PCL-5 scores and prevalence of PTSD symptoms among two groups of teenage patients with induced abortion

The overall prevalence of PTSD symptoms among all teenage females 1 month and 3 months after induced abortion was 34% (34/100) and 19% (19/100) respectively. The intervention group had significantly fewer cases with PTSD symptoms compared to the control group (24% vs. 44%, *p* = 0.035), and lower PCL-5 scores at 1 month after the operation (27.4 ± 5.4 vs. 31.8 ± 5.7, *p* < 0.001). There were no statistically significant differences in the incidence of PTSD symptoms between the two groups at 3 months after the operation, although the intervention group had a lower incidence of PTSD symptoms (14% vs. 24%, *p* = 0.203). However, the intervention group had lower PCL-5 scores 3 months after the operation (20.5 ± 7.1 vs. 25.0 ± 7.5, *p* = 0.003) (Table 2).

**Table 2:** Comparison of PCL-5 scores and prevalence of PTSD symptoms among two groups of teenage patients with induced abortion.

	Intervention group (*n* = 50)	Control group (*n* = 50)	*p*-value	OR (95%CI)
1 month after abortion				
PCL-5scores	27.4 ± 5.4	31.8 ± 5.7	<0.001 (*t* = 4.005)	
PTSD symptoms (*n*, %)	12 (24%)	22 (44%)	0.035 (*X*^2^ = 4.456)	0.402 (0.178–0.915)
3 month after abortion				
PCL-5 scores	20.5 ± 7.1	25.0 ± 7.5	0.003	
PTSD symptoms (*n*, %)	7 (14%)	12 (24%)	0.202 (*X*^2^ = 1.624)	0.516 (0.200–1.483)

### Comparison of SDS scores and prevalence of depression symptoms among two groups of teenage patients with induced abortion

The overall prevalence of depression symptoms was found to be 21% (21/100) at 1 month and 8% (8/100) at 3 months after the procedure. The intervention group exhibited a significantly lower prevalence of depression symptoms at 1 month after induced abortion (5% vs. 16%, *p* < 0.001); but no significant differences were observed at 3 months after the procedure (4% vs. 12%, *p* = 0.269). Furthermore, the intervention group showed significantly lower SDS scores compared to the control group at both 1 month and 3 months after induced abortion (31.8 ± 5.4 vs. 37.8 ± 6.6, 26.8 ± 5.0 vs. 31.4 ± 7.2, *p* < 0.05, respectively) (Table 3).

**Table 3:** Comparison of SDS scores and prevalence of depression symptoms among two groups of teenage patients with induced abortion.

	Intervention group (*n* = 50)	Control group (*n* = 50)	*p*-value	OR (95%CI)
1 month after abortion				
SDS scores	39.5 ± 6.6	46.9 ± 8.3	<0.001 (*t* = 4.878)	
Depressive symptoms (*n*, %)	5 (10%)	16 (32%)	0.007 (*X*^2^ = 7.294)	0.236 (0.089–0.696)
1 month after abortion				
SDS scores	33.1 ± 6.2	38.8 ± 8.9	<0.001 (*t* = 3.674)	
Depressive symptoms (*n*, %)	2 (4%)	6 (12%)	0.140 (*X*^2^ = 2.174)	0.306 (0.061–1.313)

### Comparison of SAS scores and prevalence of anxiety symptoms among two groups of teenage patients with induced abortion

It was found that the anxiety characteristics in teenagers with induced abortion were not significant, with an overall anxiety prevalence rate of 6% (6/100) at 1 month and 0% (0/100) at 3 months. When comparing the incidence of anxiety between the two groups, no significant difference was observed at 1 month and 3 months after induced abortion (0% vs. 6%, *p* = 0.027; 0% vs. 0%, NS). However, the intervention group displayed lower SAS scores than the control group at 1 month and 3 months after abortion (32.7 ± 5.0 vs. 39.8 ± 6.9, 25.0 ± 2.7 vs. 27.5 ± 2.8, *p* < 0.05, respectively) (Table 4).

**Table 4:** Comparison of SAS scores and prevalence of anxiety symptoms among two groups of teenage patients with induced abortion.

	Intervention group (*n* = 50)	Control group (*n* = 50)	*p*-value
1 month after abortion			
SAS scores	32.7 ± 5.0	39.8 ± 6.9	<0.001 (*t* = 5.824)
Anxiety symptoms (*n*, %)	0 (0%)	6 (12%)	0.027 (*X*^2^ = 6.383)
1 month after abortion			
SAS scores	25.0 ± 2.7	27.5 ± 2.8	<0.001 (*t* = 4.553)
Anxiety symptoms (*n*, %)	0 (0%)	0 (0%)	NS

## Discussion

Patients who undergo abortions may experience emotional distress, although research indicates that the abortion procedure itself typically does not lead to mental health issues. However, factors such as personal susceptibility and societal prejudice can contribute to negative emotions among these individuals. Consequently, many patients may experience intense negative emotions following an abortion, thereby increasing the likelihood of developing mental illness. Offering psychological guidance to patients can help alleviate their psychological burden and eliminate negative emotions. The aim of this study was to investigate the impact of intensive post-abortion care (PAC) psychological nursing on adverse emotions and post-traumatic stress disorder in female adolescent patients who have undergone induced abortion.

The incidence of PTSD following induced abortion varies significantly across different studies. A study conducted by the University of California found that 40% of women who had undergone an abortion experienced one or more symptoms of PTSD, with 16% meeting the diagnostic, criteria for PTSD (23). Another prospective study revealed that 32.5% of women exhibited symptoms of PTSD within 7 days after having an induced abortion (24). Furthermore, research on the lifetime prevalence of PTSD among different age groups indicated that the highest rates of PTSD were observed in women aged 15–24 years (25). Additionally, the risk of PTSD in adolescents who had undergone induced abortion were approximately five times more likely to develop PTSD compared to those who had not. Our study found that the overall incidence of PTSD among adolescent women was 34% at 1 month and 19% at 3 months after abortion. Several factors may contribute to the disparities in PTSD incidence between China and Western countries, including cultural variations in attitudes towards induced abortion and mental health, differences in access to mental health services, and variations in socioeconomic conditions. Age, gender, educational level, and family structure have all been identified as risk factors for the development of PTSD. Our cohort exhibited several characteristics that may explain the high incidence of PTSD. It is reported that the risk of PTSS and PTSD decreased over time among teenage women who had undergone induced abortion, which was also supported by our study. Specifically, in our study, we observed that the incidence of PTSD and the severity of symptoms at 3 months were significantly lower compared to those at 1 month. It is worth noting that appropriate psychological intervention has proven to be an effective measure in reducing or even preventing the occurrence of PTSD. Furthermore, our study revealed that the incidence of PTSD and the PCL-5 score in the intervention group, both at 1 month and 3 months after induced abortion, were significantly lower than those in the control group. Collectively, these studies highlight the effectiveness and necessity of psychological interventions for women who have undergone induced abortion, as they can help mitigate the occurrence of postoperative PTSD.

Anxiety and depression are both risk factors for PTSD. In turn, individuals with PTSD often experience co-morbid symptoms of depression and anxiety (26). Among women with PTSD, a high percentage had symptoms of anxiety (90%) and depression (76%). The incidence of depression and anxiety among teenage women who underwent induced abortion in our cohort was lower than in previous studies, with rates of 1 and 8% at 1 month, respectively. However, women with PTSD/PTSS often felt that post-abortion care was inadequate. Therefore, additional support is necessary, and simple efforts such as providing adequate pain relief, support, and privacy during this period may improve their outcomes (27). Our study demonstrated that intensive psychological intervention significantly improved the psychological well-being of post-abortion adolescent women. The intervention group exhibited lower levels of depression and anxiety symptoms at 1 month and 3 months after abortion compared to the control group, and the incidence of depression at 1 month after abortion was also lower in the intervention group. Furthermore, patients in the intervention group experienced fewer negative emotions and were able to maintain a positive attitude during the recovery period.

This study has certain limitations that should be considered. Firstly, we did not assess the baseline levels of PTSD, depression, and anxiety in the two groups of patients. However, randomization helps to guarantee that the two groups are similar in terms of characteristics such as age, gender, and incidence of PTSD, depression, and anxiety. Additionally, this study did not evaluate the likelihood of women developing lifelong PTSD, depression, and anxiety without a clinical diagnosis. Lastly, the specific source of trauma experienced by females in this study could not be determined.

In summary, intensive psychological intervention care has been shown to reduce the incidence and severity of depression, anxiety, and PTSD in teenage adolescent patients who have undergone induced abortion. The implementation of these interventions can lead to significant health benefits for teenage patients, making them highly valuable for widespread clinical application.

## Data availability statement

The raw data supporting the conclusions of this article will be made available by the authors, without undue reservation.

## Ethics statement

The studies involving humans were approved by the ethics committee of Shunde Hospital, Southern Medical University (The First People’s Hospital of Shunde). The studies were conducted in accordance with the local legislation and institutional requirements. Written informed consent for participation in this study was provided by the participants’ legal guardians/next of kin.

## Author contributions

MO designed the manuscript and enhanced the figures and language. HL, FW, GL, and SM conducted the clinical trial. HL prepared the figure and drafted this manuscript and edited and revised manuscript. All authors contributed to the article and approved the submitted version.
